# Novel peptides for kinetic studies of ligand binding to melanocortin-4 receptors using fluorescence anisotropy

**DOI:** 10.1186/2193-1801-4-S1-P23

**Published:** 2015-06-12

**Authors:** Reet Link, Sergei Kopanchuk, Ago Rinken

**Affiliations:** Institute of Chemistry, University of Tartu, Estonia; Competence Centre on Reproductive Medicine and Biology, Estonia

**Keywords:** Melanocortin-4 receptor, fluorescence anisotropy, kinetic studies

Melanocortin receptors (MCRs) are seven transmembrane G protein-coupled receptors that are known for their broad physiological relevance. The subtype 4 melanocortin receptor (MC4R) has emerged as a central element in the regulation of energy homeostasis, eating behavior and regulation of sexual functions. MCRs are governed by a complex dynamic homotropic regulation [1]. There is an increasing trend towards using fluorescence anisotropy (FA) for studying the aforementioned complex receptor-ligand interactions. FA allows the characterization of ligand binding dynamics [2]. The quality of the FA assay can be greatly increased with budded baculovirus particles that display the receptors of interest on their surfaces [3]. The use of a fluorescent ligand Cy3B-NDP-α-MSH has made it possible to study MC4Rs with higher precision and sampling rate [3]. However, this ligand has relatively slow kinetics. Modification of the structure of a MC4R antagonist revealed two new reporter ligands (UTBC101 and UTBC102) for fluorescence labeling. These new reporter ligands selectively bind to MC4Rs and exhibit improved kinetic properties. The association and dissociation rate constants of UTBC101 and UTBC102 are kon = ((2.0 ± 0.6) × 10^7 M^-1 min^-1), koff = ((4.6 ± 0.3) × 10^-3 min^-1) and kon = ((1.9 ± 0.5) × 10^7 M^-1 min^-1)), koff = ((1.0 ± 0.2) × 10^-1 min^-1), accordingly. UTBC101 and UTBC102 enable the characterization of both labelled and non-labelled ligand binding dynamics in regard to the MC4R. UTBC102 could be especially valuable for ligand screening, because of its very high dissociation rate, which makes it possible to achieve equilibrium conditions.
